# A comparison of the efficacy and safety of traditional Chinese medicine external treatment for the knee osteoarthritis: A protocol for systematic review and network meta-analysis

**DOI:** 10.1097/MD.0000000000024115

**Published:** 2021-01-08

**Authors:** Xuyu Song, Zhao Wang, Peng Zhang, Min Zhao, Lingsen Yang, Wei Zhang

**Affiliations:** a First Clinical College, Shandong University of Traditional Chinese Medicine, Jinan; b Shandong College of Traditional Chinese Medicine, Yantai; c Department of Paediatrics, Shandong Maternal And Child Health Hospital; d Department of Orthopedics, Affiliated Hospital of Shandong University of Traditional Chinese Medicine, Jinan; e Department of Orthopedics, The Third Affiliated Hospital of CCUCM, Changchun, China.

**Keywords:** KOA, network meta-analysis, protocol, traditional Chinese medicine external treatment

## Abstract

**Background:**

Knee osteoarthritis (KOA), also known as degenerative osteoarthritis, is a common and frequently occurring disease in orthopedics with cartilage degeneration as the pathogenic cause and articular bone hyperplasia as the sign. Many studies have confirmed that KOA can be effectively treated by traditional Chinese medicine (TCM) external treatment. So we take advantage of the method of network meta-analysis to systematically compare the efficacy and safety of different types of TCM external treatment for the KOA.

**Methods:**

We will research on external treatment of KOA by traditional Chinese medicine using randomized controlled trials (RCTs) in search database (EMBASE, PubMed, Web of Science, Chinese National Knowledge Infrastructure [CNKI], Weipu database [VIP], Wanfang, and China BioMedical Literature [CBM]). The data and evidence obtained will be processed using Stata 15.0 and WinBUGS 1.4.3.

**Results:**

We will evaluate the efficacy and safety of traditional Chinese medicine external treatment for the knee osteoarthritis in this study.

**Conclusion:**

This study will provide a new regimen for KOA treatment. It has extremely high reference value.

**INPLASY registration number:**

INPLASY2020120001.

**DOI number:**

: 10.37766/inplasy2020.12.0001.

## 1 Introduction

Knee osteoarthritis (KOA) is a chronic joint disease characterized by the degeneration and loss of articular cartilage of the knee joint, as well as the formation of subchondral bone and new bone at the joint margin, and characterized by joint pain, deformation, and functional limitation. According to epidemiological studies, the incidence of KOA in the United States is 12.1%, which is similar to that in Europe, and the prevalence of KOA in Canada is 10.5%. The huge number of patients has brought a heavy burden to the economic and social development.

The incidence of KOA is mostly related to factors such as old age, obesity, trauma, lifestyle, metabolism, and heredity.^[1,2]^ KOA occurs mostly in elderly patients and is an important cause of pain and disability in the elderly.^[3,4]^ And there are research data statistics, the prevalence of KOA increases with age, KOA has seriously threatened people's healthy work and life.^[5,6]^

At present, the treatment goal of Western medicine for KOA is to reduce pain and prevent joint dysfunction in various ways. The treatment methods include conservative treatment and surgical treatment. However, both methods have shortcomings.^[7,8]^ It can be said that there is no effective method to cure KOA in Western medicine.^[9]^

Although, traditional Chinese medicine (TCM) has advantages in the treatment of KOA, which belongs to the traditional dominant diseases of TCM.^[10,11]^ Acupuncture, Decoction, and external treatment are mostly used in traditional Chinese medicine to treat KOA. In recent years, relevant experimental studies of traditional Chinese medicine have also confirmed its reliable efficacy.

In this paper, we will use network meta-analysis to systematically evaluate the efficacy and safety of different kinds of TCM external treatments for KOA. This will provide a reference for future treatment.

## 2 Methods

### 2.1 Study registration

Our meta-analysis study has been registered on the International Platform of Registered Systematic Review and Meta-analysis Protocols (INPLASY). Registration number: INPLASY2020120001. DOI number: 10.37766/inplasy2020.12.0001.

### 2.2 Eligibility criteria

#### 2.2.1 Type of studies

All randomized controlled trial (RCT) studies (e.g., massage, Tai chi, yoga, five-poultry opera, etc) of TCM external treatments for KOA will be included. The language of studies is restricted to English and Chinese.^[12]^

#### 2.2.2 Types of participants

Compliance with KOA diagnostic criteria proposed by the American Rheumatology Society in 1995.^[13]^ Middle-aged and elderly patients over 40 years old.

#### 2.2.3 Interventions and comparisons

The experimental group was treated with TCM external treatments and Western medicine combined. The TCM external treatments include massage, Tai chi, yoga, and five-poultry opera, etc. The control group was treated with Western medicine only.

#### 2.2.4 Outcomes

The main evaluation indicators include Lysholm score, WOMAC score, NRS score, and Health Survey Summary Scale SF-36.

### 2.3 Search strategy

We will research on external treatment of KOA by traditional Chinese medicine using randomized controlled trials (RCTs) in EMBASE, PubMed (as shown in Table 1), Web of Science, Chinese National Knowledge Infrastructure (CNKI), Weipu database (VIP), Wanfang, and China BioMedical Literature (CBM). Furthermore, we will also search for trials that are unpublished, including the International Clinical Trials Registry Platform, the NIH Clinical Trails, and the Chinese Clinical Register.

**Table 1:** Details of the search strategy for PubMed.

No.	Search item
#1	Knee osteoarthritis[MeSH Terms]
#2	Knee osteoarthritis [Title/Abstract] OR KOA [Title/Abstract] OR osteoarthritis of knee
#3	#1 OR #2
#4	Traditional Chinese Medicine External Treatment[MeSH Terms]
#5	Exercise [Title/Abstract] OR Acupuncture [Title/Abstract] OR Moxibustion [Title/Abstract] OR Massage [Title/Abstract] OR Chinese herbal medicines [Title/Abstract] OR Yoga [Title/Abstract] OR Tai chi [Title/Abstract] OR five-poultry opera [Title/Abstract]
#6	#4 OR #5
#7	Randomized Controlled Trial[Publication Type]
#8	Randomized [Title/Abstract] OR Randomly [Title/Abstract] OR Random allocation [Title/Abstract] OR Randomized Controlled Trial[Title/Abstract]
#9	#7 OR #8
#10	#3 AND #6 AND #9

### 2.4 Data extraction

The qualified literatures in the above databases were retrieved and inserted into the EndNote X9. Two independent researchers extracted the following information from the article: fundamental information (research title, first author, sample size, age, year, course of disease, treatment period); intervention information (such as treatment method, course of treatment, comparison group, etc); key elements of bias risk evaluation; outcome indicators.

### 2.5 Risk of bias assessment

Two independent researchers used the Cochrane Risk of Bias Risk Assessment Tool to evaluate the quality of the study. There are 7 aspects to evaluate the quality of the trials, including: whether the random sequence is sufficient; whether there is hidden allocation; whether blinding is used; whether the result data is complete; whether there is selective reporting; whether there is publication bias; others.

### 2.6 Data analysis

STATA 15.0 will be used to evaluate the *P* value and *I*^2^ to quantitatively determine the size of heterogeneity. When *I*^2^ > 50%, *P* < .05, there is heterogeneity between the studies and the source of heterogeneity should be analyzed. On the contrary, there is no heterogeneity between the studies when *I*^2^ < 50%, *P* > .05.

STATA 15.0 and random-effects model will be used to conduct network meta-analysis and merge data to draw evidence network. Each effect size will be given its estimated value and 95% confidence interval. We will conduct Bayesian network meta-analysis by the Markov chain Monte Carlo method in WinBUGS 1.4.3, that is simulated by 4 chains.^[14,15]^ The trajectory of the fluctuation of the Markov chain Monte Carlo method is reflected and the potential scale reduced factor (PSRF) quantitative analysis method is used to diagnose the convergence of the model. The number of iterations and annealing times were adjusted according to the data characteristics of each outcome index and the PSRF value, and the area under the cumulative ranking probability (SUCRA) was used for ranking.

### 2.7 Assessment of heterogeneity

#### 2.7.1 Subgroup analysis

The subgroup analysis will be conducted to explore age, race, different types of TCM external treatments, treatment time, methodological quality, etc when heterogeneity is high.

#### 2.7.2 Sensitivity analysis

Some measures will be used to ensure the credibility of the research results including analysis of the same data using different statistical methods and exclusion of low-quality studies.

### 2.8 Assessment of inconsistency

When there is no statistic data difference using the node-splitting method, the direct comparison and indirect comparison are consistent.

## 3 Assessment of publication bias

We will use the funnel plot and Begger's test was used to assess the presence of publication bias.

## 4 Assessment of the quality of evidence

The grading of recommendations assessment, development, and evaluation (GRADE) method will be used to evaluate the quality of evidence. There are several aspects: risk of bias, indirectness, inconsistency, imprecision, and publication bias.

## 5 Discussion

KOA is a chronic joint disease with a high incidence. It is characterized by degeneration and loss of articular cartilage of the knee joint, as well as new bone formation in subchondral bone and joint margin, with joint pain, deformation, and functional limitation as its clinical manifestations. KOA occurs mostly in elderly patients and is an important cause of pain and disability in the elderly. With the increasing aging of the global population, KOA has seriously threatened people to work and live healthily.

The main methods treating KOA of Western medicine include oral nonsteroidal anti-inflammatory drugs and glucosamine sulfate, among others, but these methods have particular disadvantages.^[7,8]^ So, there is no effective cure for KOA in Western medicine at present. TCM treatment of KOA has a history of thousands of years, and has the advantages of remarkable curative effect and low cost. Among them, the external treatment has the characteristics of TCM, including massage, Tai chi, yoga, five-poultry opera, etc. There are many clinical studies on TCM external treatment of KOA, but few studies have compared the efficacy of different treatment methods. Therefore, the purpose of this study is to provide more convincing and detailed information for the TCM external treatment of KOA, and to provide references for the clinical treatment of such diseases. We believe that this study will attract the attention of many doctors and scholars.

## Author contributions

**Conceptualization:** Wei Zhang, Zhao Wang, Lingsen Yang, Xuyu Song.

**Data curation:** Xuyu Song.

**Formal analysis:** Peng Zhang.

**Funding acquisition:** Lingsen Yang, Wei Zhang.

**Methodology:** Xuyu Song, Zhao Wang, Peng Zhang, Min Zhao, Lingsen Yang.

**Project administration:** Lingsen Yang, Wei Zhang.

**Resources:** Lingsen Yang.

**Software:** Min Zhao.

**Supervision:** Min Zhao.

**Validation:** Lingsen Yang.

**Writing – original draft:** Lingsen Yang, Xuyu Song, Zhao Wang, Peng Zhang, Min Zhao.

**Writing – review & editing:** Lingsen Yang, Wei Zhang.
